# Engagement With a Remote Symptom-Tracking Platform Among Participants With Major Depressive Disorder: Randomized Controlled Trial

**DOI:** 10.2196/44214

**Published:** 2024-01-19

**Authors:** Katie M White, Ewan Carr, Daniel Leightley, Faith Matcham, Pauline Conde, Yatharth Ranjan, Sara Simblett, Erin Dawe-Lane, Laura Williams, Claire Henderson, Matthew Hotopf

**Affiliations:** 1 Department of Psychological Medicine Institute of Psychiatry, Psychology and Neuroscience King's College London London United Kingdom; 2 Department of Biostatistics and Health Informatics Institute of Psychiatry, Psychology and Neuroscience King's College London London United Kingdom; 3 School of Psychology University of Sussex Falmer United Kingdom; 4 Department of Psychology Institute of Psychiatry, Psychology and Neuroscience King's College London London United Kingdom; 5 NIHR MindTech MedTech Co-operative Institute of Mental Health and Clinical Neurosciences University of Nottingham Nottingham United Kingdom; 6 Health Services & Population Research Department King’s College London London United Kingdom; 7 South London and Maudsley NHS Foundation Trust London United Kingdom

**Keywords:** remote measurement, technology, engagement, app, depression, smartphones, wearable devices, engagement, symptom tracking, self-awareness, community, mobile phone

## Abstract

**Background:**

Multiparametric remote measurement technologies (RMTs), which comprise smartphones and wearable devices, have the potential to revolutionize understanding of the etiology and trajectory of major depressive disorder (MDD). Engagement with RMTs in MDD research is of the utmost importance for the validity of predictive analytical methods and long-term use and can be conceptualized as both objective engagement (data availability) and subjective engagement (system usability and experiential factors). Positioning the design of user interfaces within the theoretical framework of the Behavior Change Wheel can help maximize effectiveness. In-app components containing information from credible sources, visual feedback, and access to support provide an opportunity to promote engagement with RMTs while minimizing team resources. Randomized controlled trials are the gold standard in quantifying the effects of in-app components on engagement with RMTs in patients with MDD.

**Objective:**

This study aims to evaluate whether a multiparametric RMT system with theoretically informed notifications, visual progress tracking, and access to research team contact details could promote engagement with remote symptom tracking over and above the system as usual. We hypothesized that participants using the adapted app (intervention group) would have higher engagement in symptom monitoring, as measured by objective and subjective engagement.

**Methods:**

A 2-arm, parallel-group randomized controlled trial (participant-blinded) with 1:1 randomization was conducted with 100 participants with MDD over 12 weeks. Participants in both arms used the RADAR-base system, comprising a smartphone app for weekly symptom assessments and a wearable Fitbit device for continuous passive tracking. Participants in the intervention arm (n=50, 50%) also had access to additional in-app components. The primary outcome was objective engagement, measured as the percentage of weekly questionnaires completed during follow-up. The secondary outcomes measured subjective engagement (system engagement, system usability, and emotional self-awareness).

**Results:**

The levels of completion of the Patient Health Questionnaire-8 (PHQ-8) were similar between the control (67/97, 69%) and intervention (66/97, 68%) arms (P value for the difference between the arms=.83, 95% CI −9.32 to 11.65). The intervention group participants reported slightly higher user engagement (1.93, 95% CI −1.91 to 5.78), emotional self-awareness (1.13, 95% CI −2.93 to 5.19), and system usability (2.29, 95% CI −5.93 to 10.52) scores than the control group participants at follow-up; however, all CIs were wide and included 0. Process evaluation suggested that participants saw the in-app components as helpful in increasing task completion.

**Conclusions:**

The adapted system did not increase objective or subjective engagement in remote symptom tracking in our research cohort. This study provides an important foundation for understanding engagement with RMTs for research and the methodologies by which this work can be replicated in both community and clinical settings.

**Trial Registration:**

ClinicalTrials.gov NCT04972474; https://clinicaltrials.gov/ct2/show/NCT04972474

**International Registered Report Identifier (IRRID):**

RR2-10.2196/32653

## Introduction

### Background

Multiparametric remote measurement technologies (RMTs), which comprise smartphone apps and wearable devices, have the potential to revolutionize the clinical care of people with chronic, episodic health conditions [1]. Major depressive disorder (MDD) is one such condition, characterized by the relapse and remission of low mood and anhedonia over time [2]. Continuously measured longitudinal RMT data on the symptoms of MDD (mood variability, activity, cognition, and sleep) can capture a less biased picture of clinical state than retrospective self-report data [3]. Research using multiparametric sources might identify signals that could potentially predict future depressive episodes [4]. Such data could be ultimately implemented in patient self-management and shared decision-making in clinical practice [5].

It is important to understand how users engage with RMTs for depression symptom tracking. A recent systematic review found that engagement with RMTs can be measured objectively, for example, as the number of app-based symptom-tracking assessments completed, and subjectively, for example, as the perceived usability of and experience of using the RMT system [6]. Higher levels of objective engagement result in increased data availability, which, in turn, increases the validity of the machine learning approaches used for relapse prediction [7]. Objective engagement can also be used as an indicator of real-world uptake [8,9]. Further evidence suggests that increased satisfaction with mobile health apps is positively associated with the intention to continually use the tools [10]. Therefore, understanding engagement with RMTs is key to realizing their potential for relapse prediction.

Previous studies have reported inconsistent levels of engagement with RMT systems. Data completion, based on the total data expected, ranges from 42% to 82% for app-based symptom reporting and from 50% to 75% for device wear time [11]. The largest, multisite study of multiparametric RMTs for tracking depression to date, Remote Assessment of Disease and Relapse–Major Depressive Disorder (RADAR-MDD) [12], tracked 623 participants for 2 years using a smartphone app for mood tracking and a wrist-worn wearable for continuous passive data collection. The study has recently reported data availability metrics; 55.4% (345/623) of the sample completed >50% of the self-reported mood questionnaires expected to be completed, and 70.1% (437/623) had wearable heart rate data for >50% of the study days. Qualitative analyses from RADAR-MDD have revealed that the presence of a physical research team providing technological support and planned task reminders was a fundamental facilitator of long-term engagement in the study [13]. To ensure the scalability and real-world implementation of RMT systems, it is important to investigate methods that maximize engagement with RMTs while minimizing the human resources needed.

Focusing on the user interface (UI) of RMT systems is the logical first step for promoting engagement. Positioning the design of system UI within a theoretical framework of behavior change could help maximize effectiveness [14]. The Behavior Change Wheel [15] posits that researchers should begin by identifying a target behavior before considering the barriers to and facilitators of this behavior in terms of capability, opportunity, and motivation (the capability, opportunity, motivation, and behavior [COM-B] model). In the case of RMTs, the target behavior can be defined as objective engagement with symptom monitoring tasks. A series of published studies have evaluated both perceived [11,16] and experienced [13] barriers to RMT use in MDD research. Factors such as the knowledge of the utility of the research (capability), motivation linked to mood (motivation), and confirmation of logged data (opportunity) have been suggested to be prominent. The Behavior Change Wheel further provides a series of “intervention functions” best suited to address these factors, each with its own related behavior change techniques. With regard to RMTs, these have been suggested to be the provision of information from credible sources, visual feedback on behavior, and access to support.

The app design literature provides several options for incorporating behavior change techniques into RMT system design. First, following the Fogg behavioral model [17], push notifications can provide a trigger to perform a behavior, such as completing a monitoring task. Notifications can include tailored content, such as insights into the benefits of self-monitoring, which serves to simultaneously motivate the user to respond to the notification and engage them in future tasks [18]. Second, visual incentives, such as graphs, can be embedded into the app to reflect on patterns in user progress and spark intrinsic motivation to complete future tasks [19]. Visualization can also help users manage uncertainty by attending to information about themselves [20]. A combination of qualitative and single-arm evaluation studies supports the perceived value of data visualization [21,22] and progress viewing [19] in encouraging symptom-tracking completion. Provision of contact details directly within an app can allow the user to directly and immediately access support, if required.

Without a control group, it is difficult to quantify the effect of in-app components on engagement [23]. A randomized controlled trial (RCT) of a substance abuse tracking app [20] suggested that users were 5% more likely to self-report on a day if they received a prior notification with an inspirational quote, although these results were not statistically significant. Conversely, users were 2% less likely to self-report following the provision of personalized visual data summaries; however, this main effect was significantly moderated by the prior day task completion such that those who had not completed the previous task were 36% more likely to self-report after receiving data visualization [20]. Users receiving prompts with tailored health messages, such as those highlighting the beneficial effects of symptom monitoring, were 4% more likely to engage in self-monitoring via another app for mental well-being [24]. It is important to replicate this work with multiparametric symptom monitoring systems, as it is currently unclear which combination of in-app features best promotes engagement with these technologies.

### This Study

This study aimed to evaluate whether in-app components could promote engagement with a multiparametric RMT system for symptom tracking in depression. We conducted a 2-arm RCT to compare the system as usual with an adapted system that contained informative notifications, a visual progress report, and access to the research team contact details as a substitute for planned research team contact. We measured engagement as both objective and subjective concepts. This study had the following four specific objectives: (1) to describe data availability in an RCT of a multiparametric RMT system for tracking depression, (2) to test whether in-app components increase the rates of objective data completion, (3) to explore how in-app components influence the subjective experience of using the app, and (4) to understand how the components of the system are used by participants via process evaluation measures.

For objectives 2 and 3, we hypothesized that participants using the adapted app (intervention group) would have higher engagement in symptom monitoring, as measured by both objective engagement (completion of mood questionnaires) and subjective engagement (usability, utility, and emotional self-awareness).

## Methods

### Ethical Considerations

This study was approved by the Psychiatry, Nursing, and Midwifery Research Ethics Subcommittee at King’s College London (reference number: RESCM-20/21-21083) and registered as a clinical trial (reference number: NCT04972474). A trial protocol has been previously published [25].

### Trial Design

This was a single-center, 2-arm, parallel-group RCT (participant-blinded) with 1:1 randomization conducted in London, United Kingdom. We compared a remote symptom-tracking system (RADAR-base [26]; the control arm) with a system that contained additional in-app components (the intervention arm). Both the control and intervention arms were delivered via the RADAR-base system [26] using a smartphone app and a wearable Fitbit Charge (Fitbit Inc) device. Participants in the intervention arm had additional access to (1) theoretically informed notifications, (2) progress visualization, and (3) research team contact details through the study app. All participants were asked to use the system for 12 weeks.

Data were collected at baseline (0 weeks) and follow-up (12 weeks after randomization). Participants in both arms were sent 3 symptom-tracking tasks each week via the app; Fitbit data were collected continuously.

### Participants

All participants were recruited from the RADAR-MDD study between April and May 2021. The inclusion criteria were as follows: (1) previous participation in the RADAR-MDD study at the London site (which required experiencing at least 1 episode of MDD in the 2 years before enrollment), (2) consent to be contacted, (3) willingness and ability to continue to use an Android (Google LLC) smartphone (provided for use by RADAR-MDD; see the study by Matcham et al [3] for the full study protocol), and (4) willingness and ability to complete a remote enrollment session owing to the COVID-19 pandemic. Participants were excluded if they were diagnosed with one of the following comorbid psychiatric disorders: bipolar disorder, schizophrenia, psychosis, schizoaffective disorder, or dementia.

Potential participants were invited to take part (up to 3 invitations were sent per participant, as per ethical considerations) and subsequently checked for eligibility, both via email. If eligible, contact details were entered into the REDCap (Research Electronic Data Capture [27]) system, which emailed an automated link to the informed consent form and baseline questionnaires. After participants provided consent and completed the baseline questionnaires, they were sent a link to book an enrollment session (via email, phone call, or video call).

On the day of the enrollment session, the principal investigator (KMW) initiated the REDCap randomization module and generated unique QR codes to link the study devices to the RADAR-base management portal. Each participant was sent a personalized set of instructions for downloading and logging into the system using the QR codes at the chosen enrollment time, accompanied by a phone or video call as requested.

Participants were purposefully not contacted by the research team during the follow-up period, aside from sending 1 check-in email at the 6-week time point. However, participants were able to initiate contact with the team if they had any queries during follow-up. The research team did not make withdrawals based on “lost to follow up,” given the fundamental aims of the study; however, participants were aware that they could withdraw at any point.

Suicidal ideation was assessed at baseline and follow-up using the Inventory of Depressive Symptomatology–Self-Report [28] item “thoughts of death or suicide.” Participants who reported suicidal ideation and intent at either time point were contacted via phone call by the principal investigator, advised to contact their treating physician, and emailed a list of signposting resources.

At the 12-week end point, participants were directed to debrief information that explained the aims of the study and provided instructions for logging out of the system.

### RADAR-Base

The RADAR-base system is an open-source platform that supports data collection via remote devices [3,26]. It requires users to download and log into an Android smartphone app in addition to wearing and syncing a wearable device. All participants were asked to complete the following three validated symptom-tracking tasks per week via the study app: (1) Patient Health Questionnaire-8 (PHQ-8 [29]); (2) Rosenberg Self-Esteem Scale (RSES [30]); and (3) a speech task, during which the user records themselves reading aloud a short paragraph (Multimedia Appendix 1). All tasks became available on the same day each week, 1 hour apart, beginning at the point of enrollment. All tasks had to be completed within 24 hours.

### Interventions

#### Control Arm

Participants were sent 3 tasks per week via the RADAR-base study app, as outlined in the previous section. For each task, they received a notification on the day that the task was due that read, “Questionnaire Time. Won’t usually take longer than 3 minutes.” They were unable to view any data other than those available on the Fitbit app.

#### Intervention Arm

The design of the additional in-app components was grounded in behavioral theory and user research on the barriers to and facilitators of RMT use in patients with MDD [11,13,16]. The COM-B [31] framework of behavior change highlighted education, incentivization, and enablement as the most suitable forms of intervention function. Findings from research with users of the RADAR-base system allowed for the translation of these functions into tangible components tailored specifically to the needs and preferences of the target cohort [32]. It was decided that an engaging app should include notifications with information on symptom tracking from a credible source, behavioral feedback via progress visualization, and instant access to researcher contact details (see the study by White et al [25] and Multimedia Appendix 2 for a detailed overview of this process).

**Figure 1:**
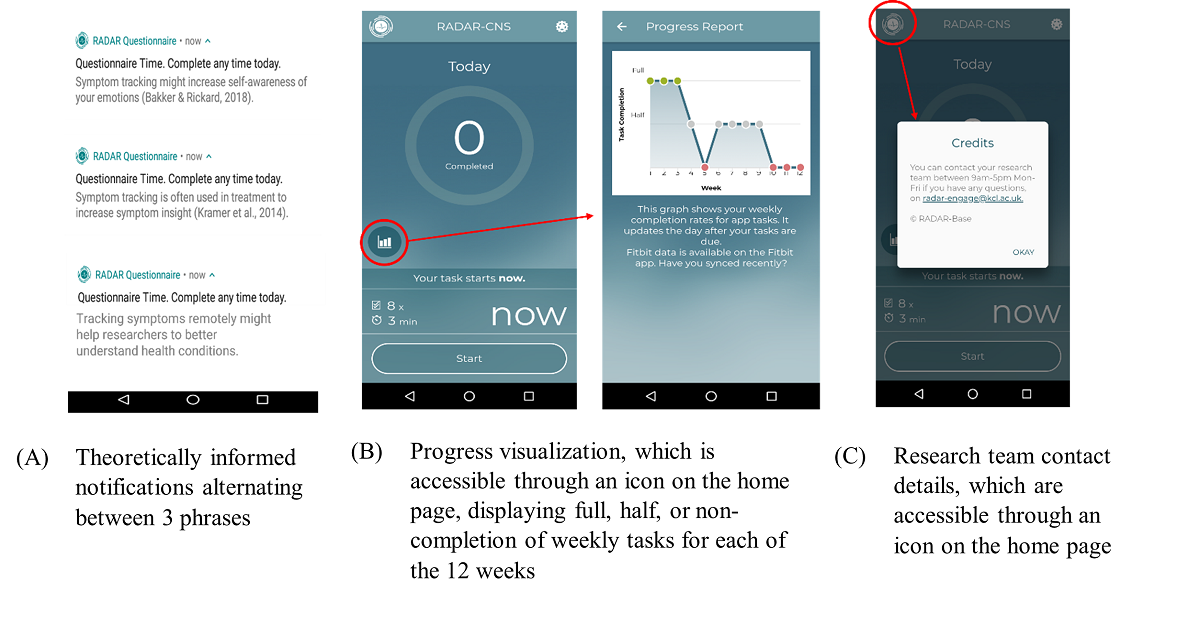
Screenshots of the in-app components included in the intervention arm.

Participants in the intervention arm received notifications and tasks at the same time as those in the control arm but with the following additional content (Figure 1):

1. Theoretically informed notifications: the notifications included additional sentences that described the potential benefits of symptom monitoring for emotional self-awareness, clinical practice, and research. Participants were also reminded that they could complete the task “any time today.”
2. Progress visualization: participants were provided with a graph in the app that tracked the completion of the tasks. This graph could be viewed at any time from the main app home page.
3. Researcher contact details: the main app home page included a phone number, an email address, and contact hours of the research team for the reporting of technical issues or requests for support.

### Measures

A summary of measures and data collection time points is presented in Table 1. The measures were identical between the intervention and control arms.

**Table 1:** A summary of measures and data collection points across the 12-week follow-up period.

Measures		Baseline	End point	Weekly	Continuously
**REDCap^a^ survey**					
	Consent	✓			
	Contact information	✓			
	Study devices	✓			
	Sociodemographics	✓			
	Social environment	✓			
	Medical history	✓			
	LIDAS^b^	✓			
	IDS-SR^c^	✓	✓		
	The World Health Organization CIDI-SF^d^	✓	✓		
	GAD-7^e^	✓	✓		
	WSAS^f^	✓	✓		
	BIPQ^g^	✓	✓		
	Life events	✓	✓		
	CSRI^h^	✓	✓		
	UES^i^	✓	✓		
	ESQ^j^	✓	✓		
	MAUQ^k^		✓		
**Active app measures**					
	PHQ-8^l^			✓	
	RSES^m^			✓	
	Speech task			✓	
**Fitbit**					
	Heart rate, step count, and GPS				✓
**Process evaluation**					
	App use metrics				✓
	Qualitative interviews		✓		

^a^REDCap: Research Electronic Data Capture.

^b^LIDAS: Lifetime Depression Assessment Self-Report.

^c^IDS-SR: Inventory of Depressive Symptomatology–Self-Report.

^d^CIDI-SF: Composite International Diagnostic Interview-Short Form.

^e^GAD-7: Generalized Anxiety Disorder-7.

^f^WSAS: Work and Social Adjustment Scale.

^g^BIPQ: Brief Illness Perception Questionnaire.

^h^CSRI: Client Service Receipt Inventory.

^i^UES: User Engagement Scale.

^j^ESQ: Emotional Self-Awareness Questionnaire.

^k^MAUQ: mHealth App Usability Questionnaire.

^l^PHQ-8: Patient Health Questionnaire-8.

^m^RSES: Rosenberg Self-Esteem Scale.

### Questionnaires

After registration for the study, participants completed web-based baseline questionnaires via REDCap, providing information on sociodemographics and physical and mental health history, including the presence of depression, recent life events, and service use. The principal investigator also manually extracted data pertaining to previous participation in the RADAR-MDD study, including participation length and technology use. At the 12-week time point, participants repeated these questionnaires.

### Outcome Measures

The primary outcome was objective engagement with the system, measured as the number and percentage of weekly PHQ-8 questionnaires completed during follow-up (compared with the total of 12 questionnaires that were sent). Completion of 1 PHQ-8 questionnaire was defined as the completion of all 8 questions.

There were four secondary outcomes, three of which measured subjective engagement with the system:

1. User engagement: this was measured using the User Engagement Scale (UES) [33] adapted to mobile health use [34], a 30-item questionnaire measuring focused attention, perceived usability, esthetic appeal, and reward. All items are scored on a Likert scale ranging from 1 (“strongly disagree”) to 5 (“strongly agree”). Total scores are calculated by summing the scores for each item in each of the 4 subscales and dividing the resultant value by the number of items in each subscale. An overall engagement score can be calculated using the average of each subscale. A higher average score indicates higher user engagement. The UES has been widely adopted and shows good reliability and construct validity [35].
2. Emotional self-awareness: this was measured using the Emotional Self-Awareness Questionnaire (ESQ) [36], a 33-item scale measuring recognition, contextualization, and decision-making in relation to self-emotion. All items are scored on a 5-point Likert scale ranging from 0 (“never”) to 4 (“a lot”). The total score is calculated as a continuous variable that ranges from 0 to 132, with a higher score reflecting higher emotional self-awareness. The ESQ has a reliability of 0.92 and shows significant positive correlations with the Emotional Intelligence Test [36].
3. System usability: this was measured using the mHealth App Usability Questionnaire (MAUQ) for stand-alone apps [37]. The MAUQ is an 18-item scale that measures the immediate and long-term self-reported usability of an app, including its ease of use and utility for self-management (overall Cronbach α=.914). All items are scored on a 7-point Likert scale ranging from 1 (“disagree”) to 7 (“agree”). The app usability score is calculated as the sum of scores across the items for each participant, ranging from 18 to 126, with a higher score reflecting higher reported usability of the app.
4. Overall adherence to the RADAR-base system: a participant was considered to have adhered to the system if they (1) responded to at least 50% of the 3 weekly tasks and (2) >2 heart rate data points were recorded by the Fitbit device on at least 50% of the days during the 12-week intervention period. This measure was chosen to align with previous data availability reporting [12] and other studies [38].

Process evaluation measures were collected to evaluate the use of the in-app components. Quantitative measures covered app engagement, in-app interactions, and notification engagement. A total of 20 participants, split evenly across the 2 arms, were also invited at the study endpoint to qualitatively discuss their experiences with the components through a 1:1 interview (Multimedia Appendix 3).

### Sample Size

Power calculations were performed based on data availability from the RADAR-MDD study [12]. To detect a difference of 25% completion of PHQ-8 tasks between the control and intervention arms, with 80% power and 95% CIs, 132 participants were required (66 per arm). We decided on 25% as the minimum difference that would be practically useful for analyses.

### Randomization and Blinding

Participants were randomly allocated in a 1:1 ratio to either the control or intervention arm using simple randomization via the REDCap randomization module.

The principal investigator was unblinded to allocation to ensure that remote enrollments had been carried out successfully and had access to incoming data throughout the study. The trial data manager (DL) was blinded to arm allocation, as this information was stored elsewhere. Participants had previously used the RADAR-base system and, therefore, could not be fully blinded to arm allocation. However, the explicit aims and arm assignments of the study were not revealed until study debrief.

### Statistical Methods

Sociodemographic and clinical variables at baseline were described by arm using appropriate summary statistics (counts and percentages for categorical variables and mean and SD or median and IQR for continuous variables). We reported data availability for all outcomes. Data availability for each study app task (PHQ-8, RSES, and speech task) was summarized as the median (IQR) number of weekly tasks completed. Fitbit wear time was summarized as mean (SD) days with >2 heart rate data points. The overall completion of all 4 data sources was also reported, calculated as a percentage of the total expected count (n=12) for the study app tasks and the total expected days of wear time (n=84) for the Fitbit.

The primary outcome, objective engagement, was analyzed using 2-sample 2-tailed *t* tests, which tested the difference in the mean percentage of PHQ-8 completion over 12 weeks between the study arms.

Three secondary outcomes (UES, ESQ, and MAUQ) were analyzed using separate linear regression models. Each model included the follow-up score as the dependent variable and arm allocation (0=control; 1=intervention) as the only covariate. Models for outcomes measured at baseline and follow-up (UES and ESQ) additionally included the baseline values of the outcomes. Differences in the combined adherence to the system (0=<50% total data completion; 1=>50% total data completion) were tested using Pearson chi-square test. The threshold for statistical significance in all the analyses was *P*=.05.

All outcomes were analyzed under the intention-to-treat principle using R (version 4.1; R Foundation for Statistical Computing) [39]. All data, including those from withdrawn participants, were included in the analyses.

### Supplementary Analysis

A supplementary analysis of all outcomes was conducted to estimate complier average causal effect (CACE) [40]. We defined compliers as participants in the intervention group who viewed the progress report page at least once in the 12 weeks. The CACE analyses were performed using a 2-stage least squares regression with arm allocation as the instrumental variable.

### Process Evaluation

We examined quantitative app use measures from data retrieved from Google Analytics (Google LLC). These were reported under the following three categories: (1) app engagement (user-initiated app opening and active weeks), (2) in-app interactions (questionnaire initiation, progress report viewing, viewed progress report >1 time, and progress report viewing duration), and (3) notification engagement (notifications received, notifications opened, and the percentage of notifications opened out of notifications received). The number of active weeks was calculated as the number of weeks the participant was active out of the total 12 weeks, with at least 3 screen view or user engagement metrics recorded per participant. Each indicator was summarized by arm as mean (SD) and median (IQR). The qualitative experiences of the study were thematically analyzed and reported as a brief narrative synthesis. These data will be reported in more detail elsewhere.

## Results

### Recruitment

**Figure 2:**
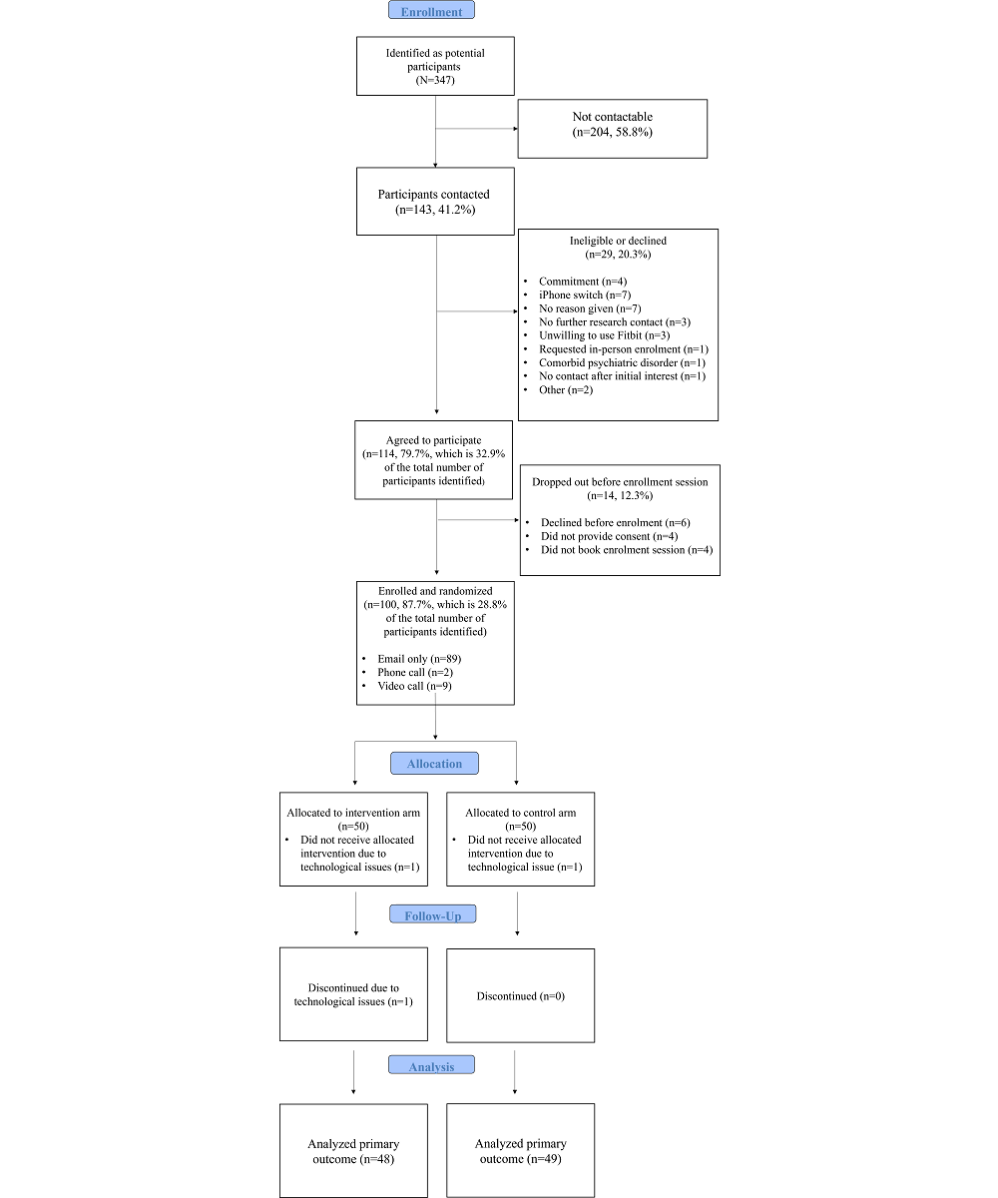
Participant flowchart following CONSORT (Consolidated Standards of Reporting Trials) guidelines.

A total of 347 individuals were contacted between April and May 2021. Of them, 114 (32.9%) agreed to participate, and 100 (28.8%) completed an enrollment session and were enrolled in the study. Enrollment sessions took place via email (89/100, 89%), video call (9/100, 9%), or phone call (2/100, 2%). Figure 2 details the participation rate and reasons for nonparticipation.

### Sample Characteristics

All (100/100, 100%) participants completed the baseline outcome assessment, and 87 (87%) participants completed the 12-week follow-up assessment. Among the total 100 participants, 1 (1%) participant in the intervention group withdrew from the study before the 12-week point, citing technological issues with the study apps as the main reason for withdrawal. The follow-up period was from April to September 2021.

Baseline characteristics were similar between the 2 groups (Table 2). The groups contained an equal number of participants (n=50). The mean age of the sample was 53.3 (SD 14.3) years, and 76 (76%) of the 100 participants were female. Most participants reported mild (36/100, 36%) or moderate (29/100, 29%) symptoms of depression at enrollment, as measured by the Inventory of Depressive Symptomatology–Self-Report. Overall, 12 (12%) participants reported suicidal ideation at baseline. Among the 100 participants, 59 (59%) “strongly agreed” that they were confident in using the smartphone they were using for the study, and 51 (51%) “strongly agreed” that they were confident in using the Fitbit device.

**Table 2:** Baseline characteristics and demographics of the study sample.

		Intervention (n=50)	Control (n=50)	Overall (N=100)
Age (y), mean (SD)		55.3 (12.7)	51.2 (15.7)	53.3 (14.3)
**Gender, n (%)**				
	Women	40 (80)	36 (72)	76 (76)
	Men	10 (20)	14 (28)	24 (24)
**Ethnicity, n (%)**				
	Black or mixed ethnicity	3 (6)	3 (6)	6 (6)
	White British	40 (80)	41 (82)	81 (81)
	White other	5 (10)	4 (8)	9 (9)
	Other	2 (4)	2 (4)	4 (4)
Total time in education (y), mean (SD)		20.2 (3.34)	20.5 (3.71)	20.4 (3.51)
**Benefit receipt, n (%)**				
	Yes	24 (48)	23 (46)	47 (47)
	No	26 (52)	27 (54)	53 (53)
**Income (£; US $), n (%)**				
	<15,000 (US $18,828.67)	9 (18)	12 (24)	21 (21)
	15,000-24,000 (US $18,828.67-$30,125.88)	8 (16)	9 (18)	17 (17)
	24,000-40,000 (US $30,125.88-$50,209.8)	15 (30)	10 (20)	25 (25)
	40,000-55,000 (US $50,209.8-$69,038.47)	11 (22)	7 (14)	18 (18)
	>55,000 (US $69,038.47)	7 (14)	12 (24)	19 (19)
**Employment status, n (%)**				
	Employed	24 (48)	25 (50)	49 (49)
	Sick leave	0 (0)	3 (6)	3 (3)
	Retired	20 (40)	14 (28)	34 (34)
	Unemployed	2 (4)	4 (8)	6 (6)
	Other	4 (8)	4 (8)	8 (8)
Current depression (continuous), mean (SD)^a^		24.8 (13.7)	26.5 (13.3)	25.7 (13.5)
**Current depression (categorical), n (%)** ^a^				
	None	10 (20)	8 (16)	18 (18)
	Mild	20 (40)	16 (32)	36 (36)
	Moderate	12 (24)	17 (34)	29 (29)
	Severe	5 (10)	7 (14)	12 (12)
	Very severe	3 (6)	2 (4)	5 (5)
**Suicidal ideation, n (%)**				
	Yes	3 (6)	9 (18)	12 (12)
	No	47 (94)	41 (82)	88 (88)
Current anxiety (continuous), mean (SD)^b^		6.34 (4.62)	7.10 (5.21)	6.72 (4.92)
**Current anxiety (categories), n (%)^b^**				
	None	22 (44)	17 (34)	39 (39)
	Mild	16 (32)	20 (40)	36 (36)
	Moderate	9 (18)	6 (12)	15 (15)
	Severe	3 (6)	7 (14)	10 (10)
**Medical comorbidity, n (%)**				
	Yes	25 (50)	34 (68)	59 (59)
	No	25 (50)	16 (32)	41 (41)
**Functional disability, n (%)**				
	No impairment	14 (28)	17 (34)	31 (31)
	Some impairment	17 (34)	17 (34)	34 (34)
	Significant impairment	19 (38)	16 (32)	35 (35)
Life events in the past year, mean (SD)		0.680 (1.04)	0.920 (1.07)	0.800 (1.05)
**Confidence in smartphone use, n (%)**				
	Strongly agree	29 (58)	30 (60)	59 (59)
	Agree	15 (30)	16 (32)	31 (31)
	Neither agree nor disagree	5 (10)	2 (4)	7 (7)
	Disagree	0 (0)	2 (4)	2 (2)
	Strongly disagree	1 (2)	0 (0)	1 (1)
**Confidence in Fitbit (Fitbit Inc) use, n (%)**				
	Strongly agree	27 (54)	24 (48)	51 (51)
	Agree	16 (32)	22 (44)	38 (38)
	Neither agree nor disagree	6 (12)	3 (6)	9 (9)
	Disagree	0 (0)	0 (0)	0 (0)
	Strongly disagree	0 (0)	0 (0)	0 (0)
	Not using Fitbit	1 (2)	1 (2)	2 (2)
**Existing RADAR-MDD^c^ status, n (%)**				
	Finished 2 years	0 (0)	2 (4)	2 (2)
	Continuing past 2 years	13 (26)	17 (34)	30 (30)
	Not reached 2 years	36 (72)	31 (62)	67 (67)
	Withdrawn	1 (2)	0 (0)	1 (1)
**Existing phone status, n (%)**				
	Existing Android (Google LLC)	30 (60)	27 (54)	57 (57)
	Switching from iPhone (Apple Inc)	13 (26)	14 (28)	27 (27)
	Switching from nonsmartphone	2 (4)	4 (8)	6 (6)
	Upgrading existing Android	5 (10)	5 (10)	10 (10)

^a^Measured using the Inventory of Depressive Symptomatology–Self-Report. The maximum score possible is 84. The scores are categorized as follows: none=0-13, mild=14-25, moderate=26-38, severe=39-48, and very severe=49-84.

^b^Measured using the Generalized Anxiety Disorder-7 item scale. The maximum score possible is 21. The scores are categorized as follows: none=0-5, mild=6-10, moderate=11-15, and severe=16-21.

^c^RADAR-MDD: Remote Assessment of Disease and Relapse–Major Depressive Disorder.

### Data Availability

Of the 100 participants, 97 (97%) provided any data via the study app, and 93 (93%) had any recorded Fitbit data. A total of 2 (2%) participants were unable to use either the study app or Fitbit immediately following enrollment, 1 (1%) received no notifications from the study app during follow-up, and 4 (4%) were unable to sync the Fitbit with their smartphone. Moreover, 1 (1%) participant opted out of using a Fitbit for the study at enrollment. As data from the sources were unavailable owing to technical limitations, rather than nonengagement, these participants were excluded from the respective analyses (3/100, 3% for primary analysis and 8/100, 8% for the secondary combined adherence analysis).

Each app task had a maximum count of 12 (1 per week). Overall, participants completed a median of 9 (IQR 6-10) PHQ-8 tasks, 9 (IQR 6-10) RSES tasks, and 8 (IQR 6-9) speech tasks. Among the 100 participants, 2 (2%) completed all available tasks, and 7 (7%) completed all available PHQ-8 tasks. A total of 35 (35%) participants completed all 3 tasks at each point when they completed a PHQ-8 task.

The participants provided sufficient Fitbit data (at least 2 recordings per day) on a mean average of 74 (SD 19.7) days per participant during the 12-week (84-day) intervention period.

**Figure 3:**
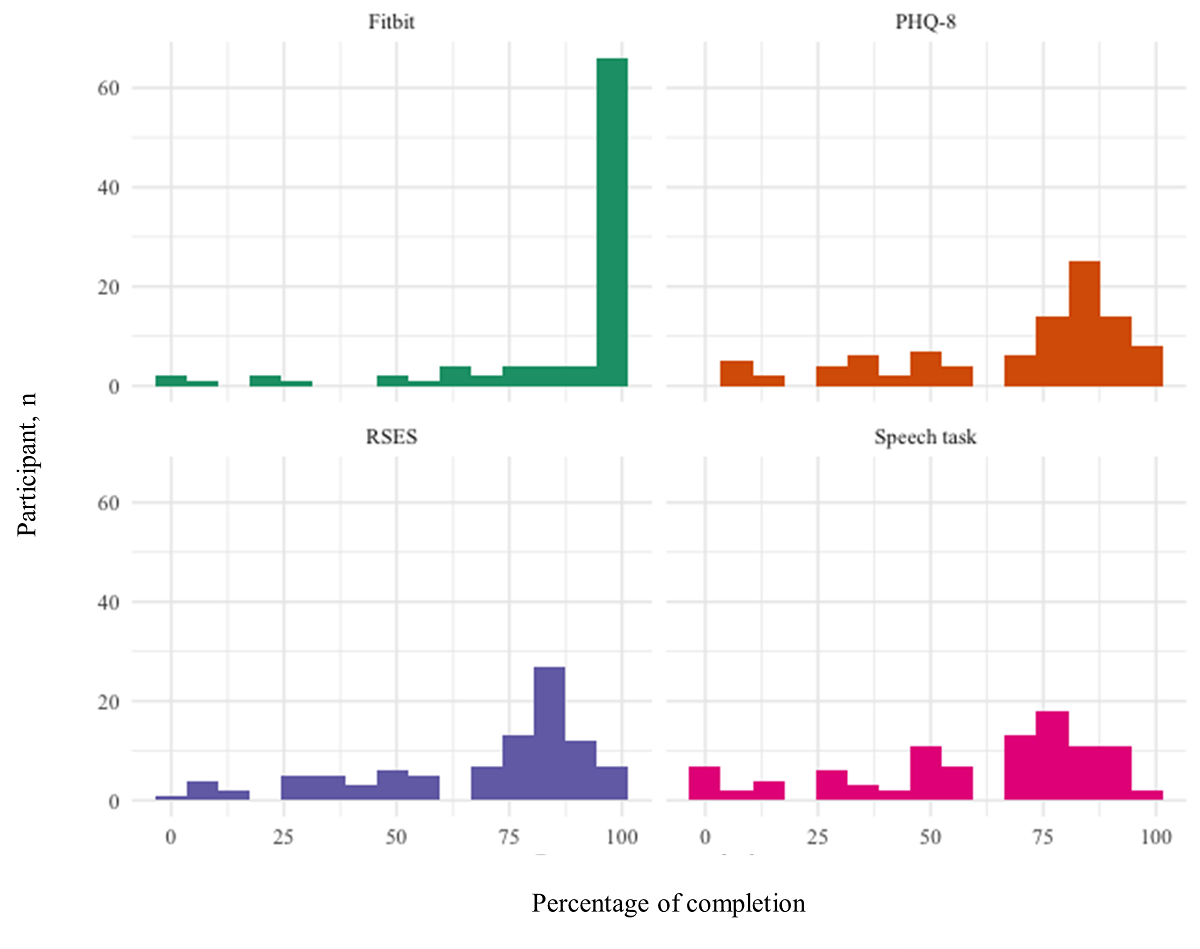
Percentage of completion for the Fitbit and the 3 active tasks (Patient Health Questionnaire-8 [PHQ-8], Rosenberg Self-Esteem Scale [RSES], and speech task).

Figure 3 shows the percentage of completion for all 4 data sources across the sample.

### Primary and Secondary Outcomes

The primary analysis included 97 participants. The levels of completion of the PHQ-8 task were similar between the control (67/97, 69%) and intervention (66/97, 68%) arms (*P* value for the difference between the arms=.83, 95% CI −9.32 to 11.65).

For the secondary outcomes (Table 3), we found that those in the intervention group reported slightly higher UES (1.93, 95% CI −1.91 to 5.78), ESQ (1.13, 95% CI −2.93 to 5.19), and MAUQ (2.29, 95% CI −5.93 to 10.52) scores than those in the control group at follow-up. However, all CIs were wide and included 0.

**Table 3:** Linear regression model coefficients for each of the 3 secondary outcomes.

Subjective engagement outcome	Treatment effect (95% CI)	Participant (N=100), n (%)^a^
UES^b^	1.93 (−1.91 to 5.78)	89 (89)
ESQ^c^	1.13 (−2.93 to 5.19)	89 (89)
MAUQ^d,e^	2.29 (−5.93 to 10.52)	87 (87)

^a^For end point measures only.

^b^UES: User Engagement Scale.

^c^ESQ: Emotional Self-Awareness Questionnaire.

^d^MAUQ: mHealth App Usability Questionnaire.

^e^Only includes end point measure.

The combined adherence secondary analysis included 92 participants. The proportion of participants adhering to the system was similar between both arms (control=32/48, 67%; intervention=35/44, 80%; *P* value for difference between arms=.98).

For the CACE analyses, of the 48 participants in the intervention group, 29 (60%) met the complier definition of viewing the progress report at least once during the intervention period (Table 4). Table 5 presents the CACE treatment estimates for the primary and secondary outcomes. The between-arm difference in PHQ-8 completion was −1.92 (95% CI −19.93 to 15.54; *P*=.83), showing no evidence of a statistical difference. The treatment effect estimates for the UES, ESQ, and MAUQ were larger than the intention-to-treat estimates in favor of the intervention, but the effect sizes remained small.

**Table 4:** Number of participants and mean percentage of completion among the control group, intervention group compliers, and intervention group noncompliers (n=97).

Study arm	Participant, n (%)	Completion (%), mean (SD)
Intervention group compliers^a^	29 (30)	75.3 (23.9)
Intervention group noncompliers^b^	19 (20)	57.0 (28.0)
Control group	49 (50)	69.2 (25.1)

^a^Viewed the progress report module at least once during the intervention period.

^b^Did not view the progress report module during the intervention period.

**Table 5:** Intention-to-treat (ITT) and complier average causal effect (CACE) treatment estimates for the primary and secondary outcomes.

Outcome	Participant, n (%)	ITT		CACE^a^	
		Treatment effect (95% CI)	*P* value	Treatment effect (95% CI)	*P* value
PHQ-8^b^	97 (100)	−1.16 (−11.65 to 9.32)	.83	−1.92 (−19.39 to 15.54)	.83
UES^c^	89 (92)	1.93 (−1.91 to 5.78)	.32	3.49 (−3.75 to 10.73)	.34
ESQ^d^	89 (92)	1.13 (−2.93 to 5.19)	.58	2.03 (−5.32 to 9.38)	.58
MAUQ^e^	87 (90)	2.29 (−5.93 to 10.52)	.58	4.21 (−10.87 to 19.28)	.58

^a^Complier average causal effect estimates of intervention group compliers, defined as those who viewed the progress report module at least once during the intervention period.

^b^PHQ-8: Patient Health Questionnaire-8.

^c^UES: User Engagement Scale.

^d^ESQ: Emotional Self-Awareness Questionnaire.

^e^MAUQ: mHealth App Usability Questionnaire.

### Process Evaluation

Table 6 presents the quantitative process evaluation measures collected throughout the study. Over the entire study period, participants in the intervention arm opened the app a mean average of 21.2 (SD 13.5) times, whereas the participants in the control group opened the app a mean average of 19.0 (SD 9.10) times. In total, 60% (35/58) of the participants who were able to view the progress report viewed it multiple times throughout the study, viewing for a mean average of 14.7 (SD 10.9) seconds per time. Participants in both groups received a similar number of notifications, although those in the control group opened a higher mean percentage (39.9, SD 25.9) of the notifications.

**Table 6:** Process evaluation use statistics by arm over the 12-week follow-up period.

				Intervention (n=48)		Control (n=49)
**App engagement,** **mean (SD)**						
	User-initiated app opening		21.2 (13.5)		19.0 (9.10)	
	Active weeks^a^		8.96 (3.14)		8.88 (2.60)	
**In-app interactions**						
	Questionnaire initiation, mean (SD)		23.6 (10.3)		25.0 (10.5)	
	**Progress report** **viewing**					
		Values, mean (SD)	3.60 (7.64)		N/A^b^	
		Values, median (IQR)	1.00 (0-2.25)		N/A	
	**Viewed progress report >1 time** **, n (%)**					
		No	19 (40)		49 (100)	
		Yes	29 (60)		N/A	
	Progress report viewing duration (seconds), mean (SD)		14.7 (10.9)		N/A	
**Notification engagement**						
	Notifications received, mean (SD)		22.0 (13.3)		22.6 (9.82)	
	Notifications opened, mean (SD)		6.58 (6.45)		8.69 (6.36)	
	Percentage of notifications opened, mean (SD)^c^		34.3 (31.8)		39.9 (25.9)	
	None received, n (%)		2 (4)		1 (2)	

^a^Calculated as the number of weeks over the 12-week period with at least 3 screen view or user engagement metrics recorded, as per Google Analytics (Google LLC) data.

^b^N/A: not applicable; participants in the control arm were unable to view the progress report.

^c^Percentage of notifications opened based on the total notifications received for each participant.

Participants generally liked the new in-app components. They felt that the progress report could motivate task completion by providing clarity that previous tasks had been successfully logged:

[It allowed me] certainly to feel more engaged and understand...how it contributes, but also to gain an overview of my own input into it, so not just I enter the data and it disappears.

P99

Most participants found the notifications somewhat informative:

The pop up things with little quotes about “doing this helps you”...yeah I liked those, I thought that was really good. You’re doing it for a purpose.

P29

However, many participants were unsure whether they had seen all the notifications that their phone had received. Some highlighted the potentially demotivating effects of the progress report, depending on previous completion:

I think it depends what mood you’re in...if I hadn’t completed everything and I wasn’t in a good mind space I could be thinking “ooh I’ve failed.”

P40

Whereas most participants agreed that the components might motivate others, the impact of the components on participants’ own task completion was more nuanced. Instead, participating in the research study seemed to be the strongest motivation for task completion:

Because I had committed to do the study it meant that I said I am going to do it so I can’t be half-hearted about it...I want to do the best I could because it was for somebody else’s use.”

P99

Many participants discussed the beneficial effects of taking part in symptom monitoring generally, such as increased awareness of their depression and communication with others. Several additional in-app components were suggested, including a direct communication channel between the app and research team.

### Harms and Protocol Violations

No adverse or serious adverse events were reported. Among the 100 participants, 1 (1%) withdrew owing to technological issues.

## Discussion

### Principal Findings

This study conducted the first, fully remote RCT of the RADAR-base symptom-tracking system to test the effect of additional in-app components, based on behavioral change theory, on objective and subjective engagement. Overall, objective engagement was high across the sample. We found that the participants who received the adapted system (incorporating theoretically informed notifications, real-time progress reports, and researcher contact details) did not show higher levels of engagement with the system than the participants who received the system as usual. Although subjective engagement (emotional self-awareness, system utility, and usability) was slightly higher in those who received the adapted app, the difference was small and did not reach statistical significance.

### Implications and Links With Previous Work

Previous research, both usability studies [19,21,22] and RCTs [20,24], has suggested that providing notifications and progress visualization can prompt objective engagement in remote symptom monitoring. We propose several explanations as to why our results did not reflect past findings.

First, our findings may reflect the sample used. Participants were recruited from a previous study that used the RADAR-base system. This meant that they had prior experience of and interest in symptom monitoring. Previous work has also highlighted the impact of the academic setting on engagement through altruistic motivations [41]. It is possible that our results reflect a ceiling effect, whereby participants in both groups were motivated to participate in the research and complete symptom monitoring regardless of the changes to the app. This is particularly apparent given that 2 of the in-app components were designed to reflect individual achievement and benefits, aspects that might not have been as relevant in this research context.

Second, the combination of in-app components used in the adapted system might not have been sufficiently tailored to the user. The development of the app was grounded in both behavioral theory [31] and user involvement [21], which suggested that viewing real-time progress and being reminded of the proposed benefits of symptom monitoring might combat the barriers to engagement. However, although these components are proposed to encourage future tracking behavior, in practice, it is unclear how they interact with the *motivation* section of the COM-B model, in this case, the low motivation linked to low mood in our cohort of people with depression. Previous work has focused on symptom tracking for substance abuse [20] or general population [18,24] cohorts, both of which might react to incentivization in different ways from those with depression. Our qualitative discussions indeed suggested that the impact of viewing data progress might be affected by individual mood and motivational fluctuations. The addition of other virtual incentives, such as gamification [42], might have been more effective in promoting engagement with the tasks here, alongside the ability to personalize which components are seen and when they are seen.

Moreover, our components were static in that they were accessible to all the participants in the intervention group at the same time and frequency. Previous work has suggested that several factors can significantly moderate the relationship between in-app components and engagement. For example, Nahum-Shani and colleagues [20] found that receiving data insights only increased the likelihood of future self-reporting in those who were not frequent users of the app, suggesting that visualizing progress is not incentivizing (or is even perhaps “irritating”) for those who are actively engaged in the task from the beginning. Several studies have found a link between notification timing and engagement [20,24], although attempts at sensor-driven notification sending based on location have so far been unsuccessful in improving data availability [43]. Taken together, this suggests that future work is needed to understand the process of interacting with in-app components in this cohort.

Third, with regard to subjective engagement, the measures used in this study might not have reflected the experience of self-monitoring in the most nuanced manner. We used previous findings [6,41] to inform our operationalization of subjective engagement with RMTs as usability (UES), emotional self-awareness (ESQ), and utility (MAUQ). Our qualitative evaluation suggested that participants generally saw the in-app components as helpful in increasing task completion, which, in turn, might have promoted the feelings of emotional self-awareness they gained from monitoring their symptoms. We also saw that participants who viewed the progress report did so for around 15 seconds at a time, often repeatedly, which suggests a sustained interest in viewing progress. Although we did not see significant differences in either objective or subjective engagement, we did see slight treatment effects for all 3 subjective measures, which were higher still when adjusting for those who viewed the progress report. It is possible that different measures might have revealed a more significant change. For example, the UES is a tool designed primarily for digital health interventions and measures concepts such as focused attention, which are not as relevant to RMTs [6]. Measures tapping into other aspects of the experience of symptom monitoring, such as being seen as an individual [44] or the provision of a safety net [45], might have provided a more detailed understanding of the interaction among the in-app components, objective engagement, and subjective engagement in the study; however, to our knowledge, these have yet to be developed.

### Strengths and Limitations

To our knowledge, this was the first study to attempt to quantify the effect of in-app components on objective and subjective engagement with a multiparametric symptom-tracking system for depression. We used an established system that was previously used to conduct the largest, longitudinal study on RMT in MDD to date [12] and demonstrated the successful transference of the system to a remote RCT design. Methodologically, this study laid the foundation for future work to measure both objective and subjective engagements with symptom-tracking devices. We used an adapted system with in-app components, which allowed for an active control group (the system as usual) and embedded data collection to reduce confounding factors associated with the delivery medium [46]. In reference to our first aim, we have shown good data availability in the first fully remote trial of the system, with 87% (87/100) of the participants completing follow-up data collection, a median of 75% completion of symptom-tracking tasks, and a mean of 74 of 84 days of wear time data without planned researcher contact.

There are several limitations to this study. First, as mentioned, the sample was previously engaged in remote symptom tracking and driven by research altruism. This allowed for the recruitment of a large sample from an established group, obtaining results quickly and efficiently. However, it is unclear how far these results might generalize to community cohorts using symptom tracking in their daily life. Second, the study was conducted during the COVID-19 lockdown periods in the United Kingdom. A combination of increased free time and interest in health tracking could have resulted in increased engagement rates. Third, despite the large sample size, the study did not reach the intended number of participants needed to achieve the optimum statistical power. Fourth, although the app design was grounded in previous research, working within the confines of an established system gave way to certain design constraints. Some additional facilitators that arose from the COM-B analysis, such as the in-app reporting of technological malfunctions, could not be included or assessed in terms of their impact on engagement.

### Avenues for Future Work

Future work should use these findings as a basis for further RCTs quantifying the effects of RMT system design on objective and subjective engagement with remote symptom tracking. Context-specific, dynamic tailoring of notifications and data insights could be key here. Although in-app components reduce the need for human resources, the impact of external factors should not be dismissed. Our system amendments did not promote engagement over and above the system as usual; future work could seek to understand how incentives such as research team support could interact with in-app components to increase engagement, such as the use of supportive chatbots [23]. Of major importance is replicating this work with different cohorts. Using the adapted system with non–help-seeking participants or those with lower technological literacy might affect the impact of the components that we tested. For example, the impact of the theoretically informed notifications might be greater in those who are less aware of the proposed benefits of symptom monitoring. Similarly, engagement with the app is likely to vary if the app is implemented in clinical practice; progress tracking and notification content might be more impactful for those who use the system for their own direct benefit. This work could also seek to complement the RCT design with additional analysis manuscripts for increased insight into the impact of UI features. For example, this could include correlational analyses of in-app component use with the measures of objective and subjective engagement or exploring whether baseline demographics are predictive of engagement in such trials. Another area for exploration is the measurement of the subjective experience of remote symptom tracking. The development of a suitable instrument that encapsulates experiential engagement would propel the understanding of the promotion of engagement across the field.

### Conclusions

This study found that a combination of informative notifications, progress visualization, and research team contact details did not increase engagement in remote symptom tracking in our research cohort. However, the system provided good data availability, and the process evaluation measures suggested that participants saw benefits in using the adapted system. We have provided the methodology and scope for future exploration in this area, as well as opportunities to replicate this work in both community and clinical cohorts to further the promotion of engagement in remote health symptom tracking for both data collection and clinical management.

## Appendix

Multimedia Appendix 1 Speech task paragraph.

Multimedia Appendix 2 Development of the adapted system based on the capability, opportunity, motivation, and behavior framework and service user research.

Multimedia Appendix 3 Semistructured interview schedules.

Multimedia Appendix 4 CONSORT checklist.

## Abbreviations

CACE: complier average causal effect
COM-B: capability, opportunity, motivation, and behavior
ESQ: Emotional Self-Awareness Questionnaire
MAUQ: mHealth App Usability Questionnaire
MDD: major depressive disorder
PHQ-8: Patient Health Questionnaire-8
RADAR-MDD: Remote Assessment of Disease and Relapse–Major Depressive Disorder
RCT: randomized controlled trial
REDCap: Research Electronic Data Capture
RMT: remote measurement technology
RSES: Rosenberg Self-Esteem Scale
UES: User Engagement Scale
UI: user interface
