# Supplementary material for: Engagement With a Remote Symptom-Tracking Platform Among Participants With Major Depressive Disorder: Randomized Controlled Trial
Source: JMIR Mhealth Uhealth. 2024 Jan 19;12:e44214. doi: 10.2196/44214 (PMC10837755; doi:10.2196/44214)
Supplement: Multimedia Appendix 1 [file mhealth_v12i1e44214_app1.docx]

Appendix 1: Speech task paragraph

**Part 1 of 3**

The North Wind and the Sun were disputing which was the stronger, when a traveller came along wrapped in a warm cloak. They agreed that the one who first succeeded in making the traveller take his cloak off should be considered stronger than the other.

**Part 2 of 3**

Then the North Wind blew as hard as he could, but the more he blew the more closely did the traveller fold his cloak around him; and at last the North Wind gave up the attempt.

**Part 3 of 3**

Then the Sun shone out warmly, and immediately the traveller took off his cloak. And so the North Wind was obliged to confess that the Sun was the stronger of the two.

(**The North Wind and the Sun, British English Version from IPA Handbook)**
