# Supplementary material for: Engagement With a Remote Symptom-Tracking Platform Among Participants With Major Depressive Disorder: Randomized Controlled Trial
Source: JMIR Mhealth Uhealth. 2024 Jan 19;12:e44214. doi: 10.2196/44214 (PMC10837755; doi:10.2196/44214)

Appendix 2: Development of the adapted system based on the COM-B framework and service user research

1. *Service user qualitative work*


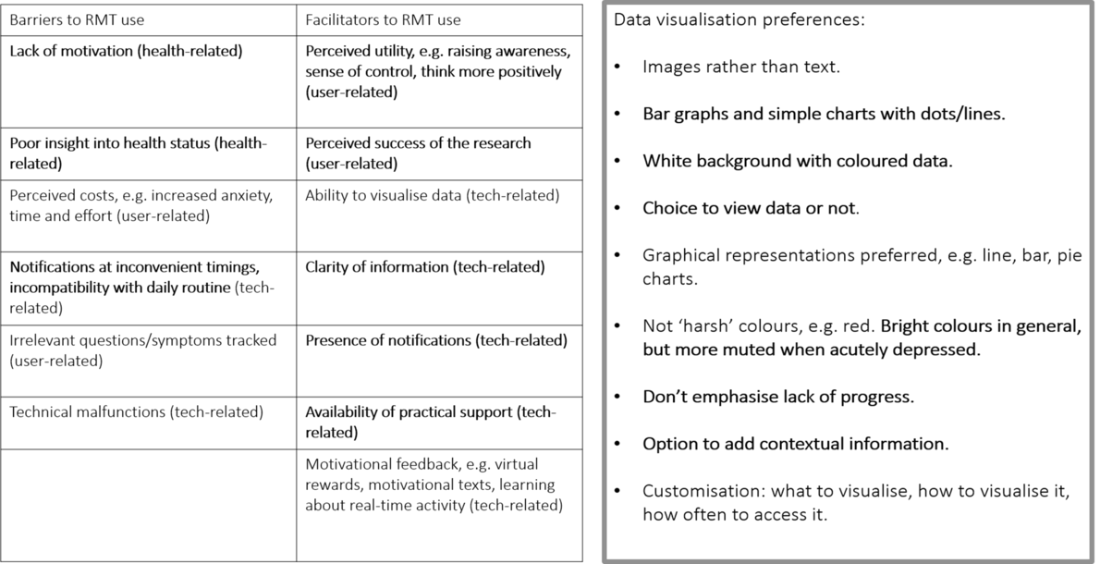


*b. COM-B model analysis, from target behavior to the in-app components.*


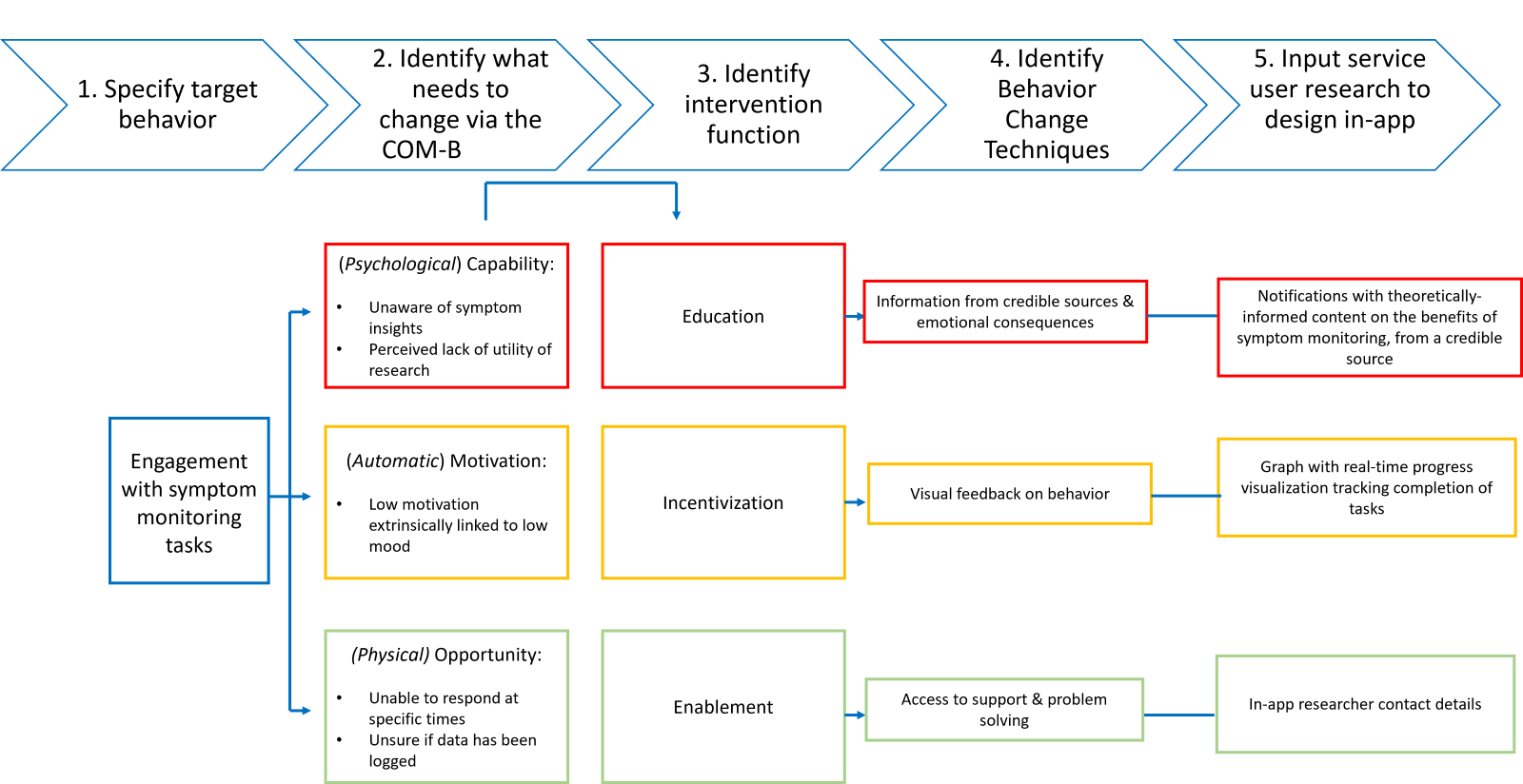

Supplement: Multimedia Appendix 2 [file mhealth_v12i1e44214_app2.docx]
