# Supplementary material for: Engagement With a Remote Symptom-Tracking Platform Among Participants With Major Depressive Disorder: Randomized Controlled Trial
Source: JMIR Mhealth Uhealth. 2024 Jan 19;12:e44214. doi: 10.2196/44214 (PMC10837755; doi:10.2196/44214)
Supplement: Multimedia Appendix 3 [file mhealth_v12i1e44214_app3.docx]

Appendix 3: Semi-structured interview schedules

Qualitative Interview Schedule (N=20)

The primary purpose of this interview is to gain insight into the effect of different in-app features on behavioral and experiential engagement with the RADAR-base system and to provide contextual information about participant experiences. The interview will be a maximum of 30 minutes and will be recorded for transcription and analyses purposes.

**Interviewer:** Thank you for participating in the RADAR: Engage study. As you might know, the study aimed to test the effect of in-app features on how participants engaged with symptom tracking through the RADAR system (the combination of smartphone apps and the Fitbit device). Engagement here was measured by the number of app questionnaires you were able to answer. We also looked at how these features affected how much you wore your Fitbit, how you felt about your symptoms, and how you found the overall usability of the system. I’d now like to ask you some questions about your experiences during the time you spent using a certain version of the app, any feedback you might have, and if and how you might envision using this technology outside of this research project. Please try to answer all questions as honestly as possible. This interview is being recorded, but you can let me know if you would like the recorder to be turned off at any point. All information you provide is anonymous- I will be writing a report as part of my PhD submission, but no identifiable information about you will be included. Do you have any questions before we begin?

| Interview Phase/Purpose | Questions/Prompts for Interviewee |
| --- | --- |
|  |  |
| **Perceptions of the trial arm the participant was randomised to**   - Understand if the participant was able to detect changes in the app | 1. How did you find your experience of participating in the study?    2. Did you find any aspect of participating in the study particularly burdensome or inconvenient?    3. As part of the study, you might have been randomized to receive a different version of the RADAR questionnaire app. Did you notice any changes to your app in comparison to when you participated in the larger study? Can you give me some examples? (LEAVE OUT FOR CONTROL)    *Prompts: Did you notice anything different about the way the app looked, or how you received reminders to answer the questionnaires?*    4. If so, do you think these had any effect on how you interacted with the app? (LEAVE OUT FOR CONTROL)    5. Is there anything about the app you used, or the system in general, that you found engaging?    6. Is there anything about the app you used, or the system in general, that you found disengaging? |
| **Experiences with the in-app techniques**   - Explore the effect of the different in-app techniques on behavioral and experiential engagement with the system | I can confirm that you were randomized to X group. This means that you [received the app as usual/received an adapted version of the app that had some changes to notifications, the data you saw and the text you saw] DELETE AS APPROPRIATE.    We are interested in your views on the effect that the different components might or might not have on your ability to interact with the app.    *For active group: [share screen to show screenshots of notification text/data visualization components]*    1a. How did you find using these new components of the app?    *Prompts: Did you view the data? Did the components provide any extra insight into the study or your participation? Did the components influence your motivation to fill out questionnaires or participate in the study?*    1b. What did you like and dislike about the features individually?    1c. Do you think these added features made any difference to the number of questionnaires you were able to answer through the app?    1d. Do you think these added features made any difference to the amount of time you wore your Fitbit?    1e. Do you think these added features made any difference to how engaged you felt with the study?    1f. Do you think these added features made any difference to how you think about the symptoms that you are tracking?    1g. Do you think these added features made you feel any differently about how you perceive your experience of depression? Did it affect your mood?    *Then, for all groups:*  Some participants received the app as usual, and some received an adapted app with different notification reminders which gave information on some of the reported benefits of tracking your symptoms, and the ability to view the progress of the completion of questionnaires on the app [share screen to show screenshot examples of the conditions]    2. Thinking about your own experience, and considering both of these components, do you think any of these are useful to have in the app?    3. Can you see any positive benefits of these components?    *Prompts: Do they make it easier to fill out questionnaires? Quicker? More enjoyable?*    4. Can you see any negative effects of these components?    *Prompts: Do they make it harder to fill out questionnaires? More time consuming? Less enjoyable?*    5. Do you think these components would affect how likely you are to engage with the symptom tracking questionnaires in different ways, or the same way? Could they work together? |
| **Areas of improvement for the questionnaire app and RADAR-base system**   - Identify opportunities for ways to further encourage engagement with symptom tracking in RMT research | 1. We have considered **three** components here that might affect how people use the RADAR system. Is there anything else you think that would make you engage more with the RADAR system?    *Prompts: Any particular in-app components? Any changes to the current ones?*    2. Is there anything that would make you engage less with the RADAR system?    *Prompts: Would these things have differential effects on answering the app questionnaires and wearing the fitbit? Is there anything outside of in-app engagement that could be done to promote or improve your engagement with the system?*    3. Would anything related to your health affect the way you participated in this study?    4. Would anything related to your experience with technology affect the way you participated in this study? |
| **The effect of symptom tracking on emotional awareness**   - Identify whether there are any correlations between objective engagement and experiential engagement | Now thinking of your experience with active and passive symptom tracking in this study **and** previously in the main RADAR study, I would like to ask you about your experiences with symptom monitoring. You were asked to monitor your symptoms for the purpose of data collection for a research study, however you may or may not have also experienced some additional effects. It’s okay if you haven’t.     1. Would you say that your understanding of your emotions has been affected by self-monitoring your symptoms?     *Prompts: In which way has this changed? Any specific symptoms, e.g., mood, self-esteem, physical activity.*     1. Would you say that your understanding of your depression has been affected by self-monitoring your symptoms?     *Prompts: In which way has this changed? Any specific symptoms, e.g., mood, self-esteem, physical activity? Has this prompted any change in your self-management or clinical care?*     1. Would you say that your experience of depression has been affected by self-monitoring your symptoms?     *Prompts: Has your experience become better? Worse? Stayed the same?* |
| **Views on using RMT systems for symptom tracking**   - Identify areas for translational work into the use of RMT systems in clinical care and self-management | So far, we have talked about engaging with symptom tracking for the purpose of a research study. I’d like to finish by talking about your views on the wider applications of symptom tracking. These could be for self-management of your condition, or for integration into clinical practice.    1. Do you see any benefits of monitoring symptoms of your depression remotely in this way? Can you give me some examples?    2. Do you see any negative effects of monitoring symptoms of your depression remotely in this way? Can you give me some examples?    *Prompts: Does answering the questionnaires or wearing the Fitbit inform you of anything to do with your condition? In what way?*    3. Do you see yourself continuing to monitor your symptoms in this way outside of this research study? Can you give me some examples? (IF MADE AVAILABLE)    *Prompts: Are there any particular parts of the RADAR system that you would or wouldn’t continue to use?*    4. Monitoring your symptoms remotely might be useful for self-management of depression. Could you see yourself monitoring your symptoms in this way? How would you do this?    5. Monitoring your symptoms remotely might also be useful for clinical care for depression. Could you see yourself or a clinician using the data in this way?    6. Is there anything that would need to be different if you continued to use the system for self-management of your condition? What about if it were to be integrated into clinical care? |
| **Closing questions and additional comments** | 1. Is there anything else that you would like to add or discuss?    Thank you very much for your time. |
